# Immunological recovery, failure and factors associated with CD-4 T-cells progression over time, among adolescents and adults living with HIV on Antiretroviral Therapy in Northern Ethiopia: A retrospective cross sectional study

**DOI:** 10.1371/journal.pone.0226293

**Published:** 2019-12-12

**Authors:** Abraham Aregay Desta, Tewolde Wubayehu Woldearegay, Asfawosen Aregay Berhe, Nesredin Futwi, Goyitom Gebremedhn Gebru, Hagos Godefay

**Affiliations:** 1 Tigray Health Research Institute, Mekelle, Tigray, Ethiopia; 2 Tigray Regional Health Bureau, Mekelle, Tigray, Ethiopia; Institut Hospital del Mar d'Investigacions Mediques, SPAIN

## Abstract

**Background:**

This study was aimed to assess immunological recovery, failure, and factors associated with CD-4 T-cells progression over time, among adolescents and adults living with HIV on Antiretroviral Therapy in Northern Ethiopia.

**Methods:**

A retrospective cross sectional study was done on 19,525 HIV patients on ART. Data were collected using a data retrieval checklist from a database. All eligible data in the database were exported to Microsoft excel 2010 and then data verification and filtration were done before exporting to STATA 14.0 for analysis. Factors associated with recent CD-4 count were modeled by using Generalized Linear Model poison family.

**Results:**

Among the patients with advanced HIV infection (< 200 CD-4 T-cell/ mm^3^) at baseline, only 28.35%, 95% CI (27.45–29.26) of them had immunological recovery (≥ 500 T-cells/mm^3^). Only 2.14%, 95%CI (1.94%- 2.35%) of the patients had immunological failure. Baseline CD-4 count (Incidence Rate Ratio (IRR) = 1.0007, 95%CI = 1.00069–1.00078), patients from military health care facility (IRR = 1.11, 95%CI = 1.06–1.16), good adherence (IRR = 1.12, 95%CI = 1.04–1.21) and viral load suppression (IRR = 1.31, 95%CI = 1.28–1.33) were positively associated with recent CD-4 count in the full model. Whereas, being male (IRR = 0.85, 95%CI = 0.83–0.86), patients with on Anti-Retroviral Therapy (ART) regimen of 1e (TDF-3TC-EFV), 2f (AZT-3TC-ATV/r), and 2h (TDF-3TC-ATV/r) (IRR = 0.92, 95%CI = 0.91–0.94), (IRR = 0.65, 95%CI = 0.55–0.76) and (IRR = 0.71, 95%CI = 0.63–0.81) respectively were negatively associated with the recent CD-4 count in the full model.

**Conclusions:**

Immunological recovery was achieved by 1/3 of the patients despite being on highly active ART (HAART). Therefore, intensive adherence counseling, follow-up and support should be focused on patients with viral non suppression to enhance immunological recovery.

## Introduction

Globally, around 36.9 million people were living with HIV and about 21.7 million people were on HAART in 2017. There were 1.8 million people who were newly infected with HIV and 940,0000 people died from Acquired Immune Deficiency Syndrome (AIDS) related illnesses in 2017 [1]. According to a study conducted in 2017/18, in urban Ethiopia, among adults aged 15–64 years, the prevalence of HIV ranged from 0.8% (in the Ethiopian Somali region) to 5.7% (in Gambella region) and the same study reported that the prevalence of HIV in Tigray region was 2.7% [2]. In a recent report, it was estimated that a total of 64,791 people were living with HIV in Tigray region [3]. Anti-Retroviral Therapy (ART) coverage in Ethiopia was moderate (52%) [4], 86% and 20% among adult and child population respectively [5]. Currently, there are 39,960 patients on ART care (61.7% coverage) in Tigray [3].

People living with HIV infection require an ongoing HIV care and access to medications to allow immune reconstitution, minimize the risk of resistance emergence [6,7], prevent HIV-related morbidity and mortality, and to prevent transmission of drug resistant HIV mutations [8, 9]. World Health Organization (WHO) recommends immunological test of individuals receiving ART to be measured every 6 months to detect immunological failure and confirm treatment failure [10, 11]. Immunological failure in adults and adolescents was defined as a CD4 count at or below 250 cells/mm3 following clinical failure or persistent CD4 levels below 100 cells/mm3 [12]. Similarly, immunological recovery was considered if the patients with advanced HIV infection (< 200 CD-4 T-cell/ mm3) at baseline have a recent CD-4 T-cells count ≥ 500 cells/mm3. In most developing countries including Ethiopia, ART initiation and monitoring was based on the WHO clinical and immunological approach [13]. Although, Viral Load (VL) determination is the golden standard to confirm treatment failure, CD-4 T-cells count can help to detect treatment failure (12).

Despite the benefits of ART, there is a growing concern about treatment failure, drug resistance and late drug toxicities associated with long-term use of ART [13, 14], mainly in eastern and southern Africa [15]. Treatment failure is related to difficulty in delivering quality ART care, and the emergence of drug resistant viruses which limits the treatment options and increases transmission, morbidity and mortality [16]. Therefore, conducting study on virological and immunological response in HIV infected patients on ART will help in continuous drug and service improvements in different settings. There was a previous study in Tigray on specific health care facilities with much smaller sample size which may not show the variability of immunological failure at different settings [17]. Thus, this study was aimed to assess immunological recovery, failure and factors associated with CD-4 T-cells progression over time, among adolescents and adults on ART living with HIV in Tigray, Northern Ethiopia. The finding of this study could help in planning to achieve the Sustainable Development Goal (SDG) to “end AIDS epidemic” by 2030.

## Methods and materials

### Study design, setting and data sources

A retrospective cross sectional study was conducted from April, 2015 to January, 2019 at Tigray Health Research Institute (THRI) which is the only center for VL determination specifically for Tigray region and some parts of Northern Ethiopia. Tigray region is the 6th largest by area and the 4th most populous of the 9 Regional States of Ethiopia [18]. The Region had an estimated population of 5,055,999 in 2016. Public health care services in Tigray are delivered through 2 specialized hospitals, 15 general hospitals, 22 primary hospitals, 202 health centers and 712 health posts. In addition, there are more than 500 private health care facilities including two general hospitals [19].

Blood samples are sent to the regional laboratory for VL determination from all the health care facilities offering HIV ART services. The sample referral form contains the following information: name of the patient, Medical Registration Number (MRN), Unique ART number, name of the health facility, some demographic data, clinical, treatment, baseline and recent CD-4 count and reason for determining viral load. The source of the data was from all the people living with HIV, enrolled in ART care for at least 6 months whose blood sample was sent for VL determination through standard sample transportation technique to the regional laboratory/THRI from April, 2015 to January, 2019. The study was done among 19525 patients which had complete data on demographic, clinical, immunological, and viral load in the database of THRI.

### Sampling procedure

**Fig 1:**
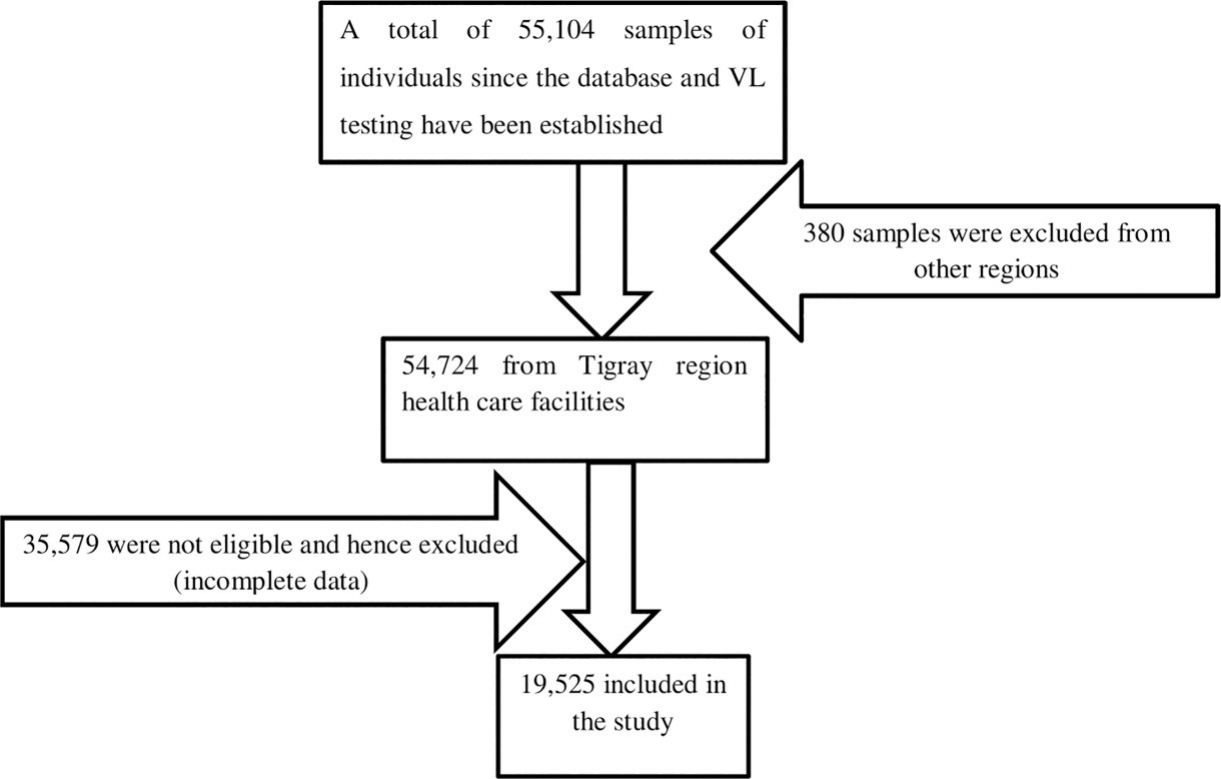
Schematic presentation of sampling procedures.

To come up with the final sample, all records of patients from the Tigray regional state available in the database of THRI were reviewed and then all the study participants which fulfilled the eligibility criteria were included in the study (Fig 1).

### Data collection tools and procedure

The data were collected from the database of THRI using a data retrieval checklist. All the data in the database were exported to Microsoft excel 2010 and then data verification and filtration were done before exporting to STATA 14.0.

CD4+ T-cells count was measured at baseline and every six months during follow up at the respective health care facilities or at the nearby referral laboratory where blood samples were transported. Nevertheless, only the results of the baseline and recent CD-4 count of each client was sent via the request form for VL determination to THRI by the health care facilities regardless of the time of initiation of ART. Hence, baseline and recent CD-4 counts were taken for analysis in this study as an independent and dependent variables respectively.

### Data quality assurance

Data completeness and consistency was checked using Microsoft excel. Data cleaning was done with box plot and running frequencies for each variable in STATA version 14.0 to check outliers and inconsistencies for accuracy purpose. The normality of the continuous variables was checked by scatter plot matrix and/or normal probability plots. Low and high positive controls were checked during VL determination. During the CD4+ T-cells count determination by each machine; low, medium and high quality controls were done to evaluate run validity in each laboratory, where the CD-4 count was done.

### Data analysis

Analysis was done using STATA-14.0 to estimate the proportion of patients with immunological failure or recovery and to identify factors associated with CD-4 T-cells progression over time. Univariate analysis was used to determine the socio-demographic and clinical characteristics of the study population.

Number of recent CD-4 T-cell counts after initiating ART was modeled by using Generalized Linear Model (GLM) with a family of Poisson with a log link function and a robust standard error. Generalized Linear Model (GLM) is an extension of the linear modeling process that allows the model to be fit to data that follow probability distributions other than the normal distribution. Poisson regression model is a special case of a generalized linear model (GLM) with a log link, the reason why the Poisson regression also called Log-Linear Model. [20]. GLM Poisson has three components; namely random, systematic and link. Poisson regression is a form of a GLM where the response variable is modeled as having a Poisson distribution. All the critical assumptions that underlie the GLM, many of which apply to any regression model: such as statistical independence of the “n” observations, the correct specification of the variance function v, the correct specification of ф (1 for Poisson and binomial data), the correct specification of the link function g, correct form of the explanatory variables x, and lack of undue influence of individual observations on the fit were fulfilled to model using Poisson GLM. Appropriate model was selected by the lowest AIC. The strength of association among the independent and dependent variables was determined by the Incidence Rate Ratio (IRR) at 95% Confidence Interval (CI). Statistical significance was considered at p-value <0.05 (two-sided) in all tests.

All significant variables in the bivariable analysis were entered into the multivariable GLM Poisson regression models based on biological plausibility, previous literature, and statistical significance in bivariable analyses. As pregnancy and lactating status applies to females, both variables were excluded from statistical model building of the multivariable analysis. Multivariable analysis was also done by stratifying the WHO stages to check whether the coefficients of variation remain constant or differ compared to the full model. Collinearity was omitted in the statistical modeling.

### Ethics statement

Ethical clearance and approval was obtained from Tigray Health Research Institute (THRI) Institutional Review Board (IRB) / Ethical Review Committee with the reference no of THRI/00132/19. Permission to use the data was obtained from the Tigray Regional Health Bureau and THRI. The data were from a secondary database on HIV infected patients for viral load monitoring while on combination of antiretroviral treatment. All the baseline and recent CD4 counts were extracted from the database retrospectively, which were entered from the sample referral form. The data were not accessible by any other third party other than the study team. All the data did not carry personal identifiers. Informed consent was waived from the ethics committee.

## Results

### Patient characteristics

A total of 19,525 patients were included in the study. The median age (IQR) of the study participants was 38 (31–45) years. Females accounted for 65.90% of the study participants. About 11,959 (61.25%) of the patients had VL determined as part of routine first VL (6 months or more on ART) determination. Only 95 (0.74%) and 202 (1.57%) were pregnant and lactating mothers respectively. A total of 18,671 (95.63%) people living with HIV were getting ART service at governmental health care facilities. Most of the patients, 18,517 (94.84%) had a good drug adherence. The median (IQR) of the baseline CD-4 T-cells count was 201 (112–341). Similarly, the median (IQR) of the recent CD-4 count was 423 (264–611). The median increase of CD-4 T-cells count from the baseline to the recent CD-4 T-cells count was 222 cells/micro Liter. The immunological response after initiation of ART based on the recent and baseline CD-4 T-cells count showed that 15,736 (80.59%) of them had positive immunological response. Most of the clients, 9,390 (48.09%) were on 1e (TDF-3TC-EFV) regimen and 19,284 (98.77%) patients were on first treatment line (Table 1).

**Table 1:** Univariate analysis of patients on ART in Tigray region, Northern Ethiopia, April, 2015 to January, 2019 (n = 19525).

Variable	Category	Frequency (Percentage)/ Median(IQR)
Gender	Female	12866 (65.90)
	Male	6659 (34.10)
Median age (IQR)		38 (31–45)
Age category in years	15–19	420 (2.15)
	20–24	555 (2.84)
	25–29	1832 (9.38)
	30–34	3716 (19.03)
	35–39	4,230 (21.66)
	40–44	3713 (19.02)
	45–49	2148 (11.00)
	50+	2911 (14.91)
Pregnant mothers	No	12771 (99.26)
	Yes	95 (0.74)
Lactating mothers	No	12664 (98.43)
	Yes	202 (1.57)
Facility ownership under care	Government	18671 (95.63)
	Non-governmental organization	840 (4.30)
	Private	14 (0.07)
Facility type	Clinic	43 (0.22)
	Health center	5682 (29.10)
	Primary hospital	2131 (10.91)
	General hospital	9829 (50.34)
	Referral Hospital	1499 (7.68)
	Other	341 (1.75)
A service provided at military health care facility	No	18850 (96.54)
	Yes	675(3.46)
WHO Stage	I	17829 (91.31)
	II	775 (3.97)
	III	408 (2.09)
	IV	513 (2.63)
Adherence	Poor	286 (1.46)
	Fair	722 (3.70)
	Good	18517 (94.84)
Baseline CD4 T-cells count	Median (IQR)	201 (112–341)
Baseline CD 4 T-cells count category	<200 cells/micro liter	9687 (49.61)
	200–499 cell/ micro liter	7605 (38.95)
	+500 cells/ micro liter	2233 (11.44)
Recent CD4 T-cells count	Median (IQR)	423 (264–611)
Recent CD4 T-cells count category	<200 cells/micro liter	3043 (15.59)
	200–499 cell/ micro liter	8909 (45.63)
	+500 cells/ micro liter	7573 (38.79)
Immunological response after ART initiation	Deteriorated	3539 (18.13)
	The same	250 (1.28)
	Positively responded	15736 (80.59)
Test reason for VL determination	Routine first VL (6 Months or more on ART)	11,959 (61.25)
	Routine annual VL test	6032 (30.89)
	Suspected ART failure clinical	50 (0.26)
	Suspected ART failure immunological	81 (0.41)
	Suspected ART failure initial VL	1091 (5.59)
	Not indicated in the form	312 (1.60)
Regimen	1c (AZT-3TC-NVP)	5832 (29.87)
	1d (AZT-3TC-EFV)	1907 (9.77)
	1e (TDF-3TC-EFV)	9390 (48.09)
	1f (TDF + 3TC+ NVP)	2142 (10.97)
	1g (ABC + 3TC + EFV)	6 (0.03)
	1h (ABC + 3TC NVP)	7 (0.04)
	2a (ABC-ddl-LPV/R), 2c (TDF-ddl-LPV/R), 2d(TDF-ddl-NFV) & 2g (TDF-3TC-LPV/r)	12 (0.06)
	2f (AZT-3TC-ATV/r)	62 (0.32)
	2h (TDF-3TC-ATV/r)	167 (0.86)
Treatment line	First line	19284 (98.77)
	Second line	241 (1.23)
Viral suppression	Suppressed	14372 (73.61)
	Non suppressed	5153 (26.39)

### Immunological recovery and failure

Among the patients with advanced HIV infection (< 200 CD-4 T-cells / mm3) at baseline, only 28.35% of them had immunological recovery (≥ 500 CD-4 T-cells /mm3). Based on the WHO definition, only 2.14%, 95%CI (1.94%- 2.35%) of the patients had immunological failure (Table 2).

**Table 2:** Immunological recovery and failure among patients on ART from April, 2015 to January, 2019 in Tigray region, Northern Ethiopia.

Characteristics	Category	Frequency (%)	95% CI
Immunological recovery among patients with advanced HIV infection, n = 9687	<200 CD-4 T-cells / mm^3^	2154 (22.24)	21.41–23.08
	200–499 CD-4 T-cells / mm^3^	4787 (49.42)	48.42–50.42
	+500 CD-4 T-cells / mm^3^	2746 (28.35)	27.45–29.26
Immunological failure. n = 19525	WHO definition 2013	3792 (19.42)	18.87–19.98
	WHO definition 2016	417 (2.14)	1.94–2.35

#### Factors associated with CD-4 T-cells progression over time

**Bivariable analysis.** The bivariable analysis showed that the Incidence Rate Ratios (IRRs) of the independent variables were associated with CD-4 T-cells progression over time. At bivariable analysis level, CD-4 T-cells count at baseline (IRR = 1. 000784, 95%CI = 1.0007–1.0008), Males (IRR = 0.79, 95%CI = 0.78–0.81), and lactating mothers (IRR = 1.08. 95%CI = 1.01, 1.16) were significantly associated with the recent CD-4 T-cells count. Similarly, patients’ age categories were significantly associated with the recent CD-4 T-cells count. However being pregnant was not statistically associated with the recent CD-4 T-cells count over time (Table 3).

**Table 3:** Bivariable analysis of variables with CD-4 T-cells count progression over time, among HIV infected patients on ART from April, 2015 to January, 2019 in Tigray region, Northern Ethiopia (n = 19525).

Variables	Category	Crude IRR (95% CI)	P-value
CD4 baseline		1.000784 (1.0007, 1.0008)	0.000
Gender	Female	1 (Ref.)	
	Male	0.79 (0.78, 0.81)	0.000
Pregnant mothers (n = 12866)	No	1 (Ref.)	
	Yes	1.07 (0.97, 1.19)	0.194
Lactating mothers (n = 12866)	No	1 (Ref.)	
	Yes	1.08 (1.01, 1.16)	0.022
Age category	15–19	1 (Ref.)	
	20–24	0.88 (0.82, 0.95)	0.001
	25–29	0.82 (0.77, 0.87)	0.000
	30–34	0.81 (0.77, 0.87)	0.000
	35–39	0.76 (0.72, 0.81)	0.000
	40–44	0.72 (0.68, 0.76)	0.000
	45–49	0.70 (0.66, 0.74)	0.000
	50+	0.69 (0.65, 0.73)	0.000
Facility ownership	Government	1 (Ref.)	
	Non-governmental organization	1.02 (0.98, 1.06)	0.432
	Private	1.21 (0.94, 1.56)	0.131
Facility type	Clinic	1 (Ref.)	
	Health center	0.82 (0.72, 0.94)	0.003
	Primary hospital	0.84 (0.73, 0.96)	0.01
	General hospital	0.91 (0.80, 1.04)	0.165
	Referral Hospital	0.79 (0.69, 0.91)	0.001
	Other	1.01 (0.87, 1.17)	0.881
A service provided at military health care facility	No	1 (Ref.)	
	Yes	0.92 (0.88, 0.95)	0.000
WHO Stage	I	1 (Ref.)	
	II	0.86 (0.82, 0.91)	0.000
	III	0.78 (0.73, 0.83)	0.000
	IV	0.98 (0.93, 1.03)	0.423
Adherence	Poor	1 (Ref.)	
	Fair	1.07 (0.97, 1.17)	0.186
	Good	1.32 (1.22, 1.43)	0.000
Virological test reason	Routine first VL (6 Months or more on ART)	1 (Ref.)	
	Routine annual VL test	1.02 (1.001, 1.04)	0.041
	Suspected ART failure clinical	0.64 (0.51, 0.80)	0.000
	Suspected ART failure immunological	0.46 (0.37, 0.56)	0.000
	Suspected ART failure initial VL	0.67 (0.65, 0.70)	0.000
	Not indicated in the form	0.93 (0.87, 0.998)	0.042
Regimen	1c (AZT-3TC-NVP)	1 (Ref.)	
	1d (AZT-3TC-EFV)	0.97 (0.94, 0.999)	0.047
	1e (TDF-3TC-EFV)	0.97 (0.95, 0.98)	0.000
	1f (TDF + 3TC+ NVP)	0.98 (0.96, 1.01)	0.261
	1g (ABC + 3TC + EFV)	0.96 (0.70, 1.31)	0.778
	1h (ABC + 3TC NVP)	0.69 (0.44, 1.09)	0.114
	2a (ABC-ddl-LPV/R), 2c (TDF-ddl-LPV/R), 2d(TDF-ddl-NFV)& 2g (TDF-3TC-LPV/r)	0.68 (0.43, 1.08)	0.101
	2f (AZT-3TC-ATV/r)	0.59 (0.50, 0.71)	0.000
	2h (TDF-3TC-ATV/r)	0.65 (0.57, 0.74)	0.000
Treatment line	First line	1 (Ref.)	
	Second line	0.65 (0.0.59, 0.72)	0.000
Viral load suppression	No	1 (Ref.)	
	Yes	1.40 (1.36, 1.43)	0.000

Patients cared in health centers (IRR = 0.82, 95%CI = 0.72–0.94), patients served in a military health care facility (IRR = 0.92, 95%CI = 0.88–0.95), Patients with a recent WHO stage II (IRR = 0.86, 95%CI = 0.82–0.91) and patients with poor adherence (IRR = 1.32, 95%CI = 1.22–1.43) were significantly associated with the recent CD-4 T-cells count. Patients with a virological test reason of suspected ART failure clinical (IRR = 0.64, 95%CI = 0.51–0.80), patients with a current regimen of 2f (IRR = 0.59, 95%CI = 0.50–0.71), Patients with a 2^nd^ treatment line (IRR = 0.65, 95%CI = 0.59–0.72) were significantly associated with the recent CD-4 T-cells count. Similarly, patients with VL suppression (IRR = 1.40, 95%CI = 1.36–1.43) were significantly associated with the recent CD-4 T-cells count. However, patients with a regimen of 1f were not significantly associated with the recent CD-4 T-cells count (Table 3).

### Multivariable analysis

A multivariable analysis using the GLM showed that the initial model without any predictor variables have an AIC of 159.3391. The constant was significant with p-value< 0.01 (Table 4).

**Table 4:** Variability of constant of the null model for recent CD-4 T-cells count among HIV infected patients on ART from April, 2015 to January, 2019, Tigray region, Northern Ethiopia.

Recent CD-4 count	IRR	Standard Error	Z	P>z	[95% Conf. Interval]
Constant	462.7631	1.918142	1480.64	0.000	459.02–466.54

After including all predictors to explain the variability of CD-4 T-cells count in the final model, the AIC has decreased from 159.3391 at the null model to 126.0423. The multivariable analysis showed that, as CD-4 T-cells count increases by one unit, the recent CD-4 T-cells count increases by a factor (IRR = 1.0007, 95%CI = 1.00069–1.00078) while holding all other variables in the model constant. Males compared to females, while holding other variables constant in the model, are expected to have a rate (IRR = 0.85, 95%CI = 0.83–0.86) times lower for the recent CD-4 T-cells count. Age category in 20–24 years compared to 15–19 years, while holding the other variables constant in the model, are expected to have a rate (IRR = 0.84, 95%CI = 0.78–0.89) times lower for the recent CD-4 T-cells count (Table 5).

**Table 5:** Multivariable analysis of the independent variables with the recent CD-4 T-cells progression over time, among HIV infected patients on ART from April, 2015 to January, 2019 in Tigray region, Northern Ethiopia (n = 19525).

Variables	Category	Adjusted IRR (95% CI)	P-value
Baseline CD4 T-cells count/mm^3^		1.0007 (1.00069, 1.00078)	0.000
Gender	Female	1 (Ref.)	
	Male	0.85 (0.83, 0.86)	0.000
Age category	15–19	1 (Ref.)	
	20–24	0.84 (0.78, 0.89)	0.000
	25–29	0.79 (0.75, 0.84)	0.000
	30–34	0.80 (0.76, 0.85)	0.000
	35–39	0.77 (0.73, 0.82)	0.000
	40–44	0.76 (0.72, 0.80)	0.000
	45–49	0.75 (0.71, 0.79)	0.000
	50+	0.73 (0.69, 0.77)	0.000
Facility type	Clinic	1 (Ref.)	
	Health center	0.94 (0.86, 1.03)	0.178
	Primary hospital	1.02 (0.93, 1.12)	0.656
	General hospital	1.12 (0.98, 1.22)	0.17
	Referral Hospital	1.00 (0.91, 1.10)	0.975
	Other	1.18 (1.07, 1.31)	0.001
A service provided at military health care facility	No	1 (Ref.)	
	Yes	1.11 (1.06, 1.16)	0.000
WHO Stage	I	1 (Ref.)	
	II	0.92 (0.89, 0.96)	0.000
	III	0.89 (0.84, 0.95)	0.000
	IV	1.00 (0.95, 1.04)	0.923
Adherence	Poor	1 (Ref.)	
	Fair	0.98 (0.90, 1.06)	0.602
	Good	1.12 (1.04, 1.21)	0.002
Virological test reason	Routine first VL (6 Months or more on ART)	1 (Ref.)	
	Routine annual VL test	1.01 (1.00, 1.03)	0.085
	Suspected ART failure clinical	0.69 (0.58, 0.81)	0.000
	Suspected ART failure immunological	0.55 (0.45, 0.66)	0.000
	Suspected ART failure initial VL	0.80 (0.77, 0.83)	0.000
	Not indicated in the form	0.96 (0.91, 1.02)	0.161
Regimen	1c (AZT-3TC-NVP)	1 (Ref.)	
	1d (AZT-3TC-EFV)	1.00 (0.98, 1.03)	0.845
	1e (TDF-3TC-EFV)	0.92 (0.91, 0.94)	0.000
	1f (TDF + 3TC+ NVP)	0.99 (0.97, 1.02)	0.453
	1g (ABC + 3TC + EFV)	1.03 (0.82, 1.29)	0.817
	1h (ABC + 3TC NVP)	0.80 (0.57, 1.12)	0.19
	2a (ABC-ddl-LPV/R), 2c (TDF-ddl-LPV/R), 2d(TDF-ddl-NFV)& 2g (TDF-3TC-LPV/r)	0.73 (0.53, 1.01)	0.06
	2f (AZT-3TC-ATV/r)	0.65 (0.55, 0.76)	0.000
	2h (TDF-3TC-ATV/r)	0.71 (0.63, 0.81)	0.000
Treatment line	First line	1 (Ref.)	
	Second line	Omitted due to collinearity	
Viral load suppression	No	1 (Ref.)	
	Yes	1.31 (1.28, 1.33)	0.000

Patients from military health care facilities compared to patients with non-military health care facilities, while holding the other variables constant in the model, are expected to have a rate (IRR = 1.11, 95%CI = 1.06–1.16) times greater for the recent CD-4 T-cells count. Patients in WHO stage II and III compared to WHO stage I, while holding the other variables constant in the model, are expected to have a rate (IRR = 0.92, 95%CI = 0.89–0.96) and (IRR = 0.89, 95%CI = 0.84–0.95) times lower for the recent CD-4 T-cells count respectively. Patients with good adherence compared to poor adherence level, while holding the other variables constant in the model, are expected to have a rate (IRR = 1.12, 95%CI = 1.04–1.21) times greater for the recent CD-4 T-cells count respectively (Table 5).

On the other hand, patients whose viral load was determined for suspected ART failure initial VL compared to routine first VL is expected to have a rate (IRR = 0.69, 95%CI = 0.58–0.81) times lower for the recent CD-4 T-cells count. Patients with a regimen 1e (TDF-3TC-EFV) compared to a 1c (AZT-3TC-NVP) regimen, while holding the other variables constant in the model, are expected to have a rate (IRR = 0.92, 95%CI = 0.91–0.94) times lower in the recent CD-4 T-cells count. Similarly, patients with viral load suppression compared to patients with virological non suppression, while holding the other variables constant in the model, are expected to have a rate (IRR = 1.31, 95%CI = 1.28–1.33) times greater for the recent CD-4 T-cells count (Table 5).

We made a stratified analysis by WHO stage to find out whether the strength of association of independent variables with the recent CD-4 T-cells count remain same or differ. Based on the analysis, baseline CD-4 T-cells count and VL suppression were positively associated and remain the same with the recent CD-4 T-cells count in all the WHO stages compared to the full model (without stratification by the WHO stage). However, being male in gender, age category in 40–44 and 45–49, and suspected ART failure initial VL for VL determination reason were negatively associated and remain almost the same with the recent CD-4 T-cells count in all the WHO stages. Despite these variables, there were differences after the stratified analysis in variables such as: age categories, service provided in military health care facility, facility type, adherence, in some of the virological test reasons and in some of the regimen types. Service provide in military health care facility was only significant in the WHO stage I sub cluster analysis (Table 6).

**Table 6:** Multivariable GLM Poisson sub-group analysis on recent CD-4 T-cells count by WHO stage among HIV patients on ART from April, 2015 to January, 2019 in Tigray region, Northern Ethiopia (n = 19525).

Variable	Category	WHO stage			
		Stage I (n = 17829)	Stage II (n = 775)	Stage III (n = 408)	Stage IV (n = 513)
		AIRR (95% CI)	AIRR (95% CI)	AIRR (95% CI)	AIRR (95% CI)
Baseline CD-4 T-cells count/ mm^3^		1.00072 (1.001, 1.001)***	1.000872(1.000667 1.001077)***	1.0009 (1.00059, 1.0006)***	1.0007 (1.0004, 1.001)***
Gender	Female	1 (Ref.)	1 (Ref.)	1 (Ref.)	1 (Ref.)
	Male	0.85 (0.83, .86)***	0.81 (0.74, 0.88) ***	0.75 (0.66, 0.86)***	0.86 (0.78, 0.96)**
Age category in years	15–19	1 (Ref.)	1 (Ref.)	1 (Ref.)	1 (Ref.)
	20–24	0.84 (0.78, 0.90)***	0.77 (0.55, 1.09)	0.47 (0.30, 0.74)**	0.92 (0.59, 1.43)
	25–29	0.80 (0.75, 0.85)***	0.74 (0.55, 0.996)*	0.67 (0.48, 0.95)*	0.75 (0.54, 1.03)
	30–34	0.80 (0.76, 0.85)***	0.71 (0.53, 0.95)*	0.74 (0.53, 1.04)	0.83 (0.64, 1.09)
	35–39	0.78 (0.73, 0.82)***	0.76 (0.57, 1.02)	0.69 (0.50, 0.96)*	0.72 (0.55, 0.94)**
	40–44	0.76 (0.72, 0.81)***	0.73 (0.55, 0.98)*	0.70 (0.50, 0.97)*	0.65 (0.49, 0.86)**
	45–49	0.75 (0.71, 0.79)***	0.71 (0.53, 0.96)*	0.67 (0.47, 0.95)*	0.75 (0.56, 0.997)*
	50+	0.73 (0.69, 0.77)***	0.67 (0.50, 0.90)**	0.73 (0.53, 1.003)	0.75 (0.56, 0.99)*
Facility ownership	Government	1 (Ref.)	1 (Ref.)	1 (Ref.)	1 (Ref.)
	NGO	0.95 (0.91, 0.98)**	0.81 (0.58, 1.12)	0.93 (0.62, 1.39)	0.88 (0.71, 1.10)
	Private	0.97 (0.80, 1.18)	--	---	---
Facility type	Clinic	1 (Ref.)	1 (Ref.)	1 (Ref.)	1 (Ref.)
	HC	0.92 (0.84, 1.01)	1.80 (1.46, 2.20)***	--	---
	PH	0.99 (0.91, 1.09)	2.04 (1.66, 2.51)***	1.11 (0.91, 1.36)	1.00 (0.77, 1.30)
	GH	1.10 (1.01, 1.20)*	2.12 (1.72, 2.60)***	0.96 (0.77, 1.20)	1.10 (0.90, 1.34)
	Referral Hospital	0.98 (0.89, 1.08)	1.87 (1.37, 2.54)***	1.28 (0.80, 2.05)	0.90 (0.64, 1.26)
	Other	1.23 (1.10, 1.36)***	2.07 (1.47, 2.92)***	0.69 (0.40, 1.20)	1.33 (0.96, 1.84)
A service provided in military health care facility	No	1 (Ref.)	1 (Ref.)	1 (Ref.)	1 (Ref.)
	Yes	1.09 (1.04, 1.14)***	1.21 (0.93, 1.57)	1.07 (0.67, 1.71)	1.23 (0.89, 1.70)
Adherence	Poor	1 (Ref.)	1 (Ref.)	1 (Ref.)	1 (Ref.)
	Fair	0.97 (0.88, 1.06)	0.92 (0.69, 1.23)	1.003 (0.74, 1.35)	1.38 (0.79, 2.40)
	Good	1.13 (1.04, 1.22)**	0.93 (0.73, 1.29)	1.00 (0.78, 1.28)	1.72 (1.01, 2.94)*
Virological test reason	Routine first VL (6 Months or more on ART)	1 (Ref.)	1 (Ref.)	1 (Ref.)	1 (Ref.)
	Routine annual VL test	1.02 (1.001, 1.03)*	1.08 (0.98, 1.20)	0.89 (0.76, 1.03)	0.91 (0.83, 1.01)
	Suspected ART failure clinical	0.66 (0.53, 0.82)***	0.66 (0.52, 0.84)**	1.32 (0.83, 2.09)	0.65 (0.40, 1.04)
	Suspected ART failure immunological	0.57 (0.46, 0.70)***	0.64 (0.40, 1.04)	0.35 (0.21, 0.59)***	0.60 (0.24, 1.54)
	Suspected ART failure initial VL	0.80 (0.77, .84)***	0.85 (0.74, 0.98)*	0.75 (0.60, 0.92)**	0.70 (0.54, 0.91)**
	Not indicated in the form	0.97 (0.92, 1.03)	0.76 (0.58, 1.01)	0.80 (0.53, 1.23)	0.61 (0.49, 0.76)***
Regimen	1c (AZT-3TC-NVP)	1 (Ref.)	1 (Ref.)	1 (Ref.)	1 (Ref.)
	1d (AZT-3TC-EFV)	1.01 (0.99, 1.04)	0.90 (0.79, 1.03)	0.92 (0.75, 1.12)	0.90 (0.75, 1.07)
	1e (TDF-3TC-EFV)	0.93 (0.91, 0.95)***	0.94 (0.85, 1.04)	0.78 (0.67, 0.90)**	0.88 (0.79, 0.99)*
	1f (TDF + 3TC+ NVP)	1.00 (0.97, 1.02)	0.97 (0.83, 1.12)	0.74 (0.60, 0.91)**	1.00 (0.88, 1.14)
	1g (ABC + 3TC + EFV)	1.15 1.08, 1.24***	---	0.29 (0.24, 0.35)***	---
	1h (ABC + 3TC NVP)	0.89 (0.63, 1.24)	---	0.28 (0.21, 0.36)***	0.74 (0.54, 1.02)
	2a (ABC-ddl-LPV/R), 2c (TDF-ddl-LPV/R), 2d(TDF-ddl-NFV)& 2g (TDF-3TC-LPV/r)	0.82 (0.60, 1.13)	---	----	0.39 (0.16, 0.96)*
	2f (AZT-3TC-ATV/r)	0.69 (0.58, 0.81)***	0.46 (0.23, 0.89)*	0.17 (0.09, 0.34)***	0.41 (0.21, 0.80)**
	2h (TDF-3TC-ATV/r)	0.72 (0.63, 0.83)***	0.69 (0.47, 1.02)	0.98 (0.63, 1.53)	0.60 (0.41, 0.88)**
Treatment line	First line	1 (Ref.)	1 (Ref.)	1 (Ref.)	1 (Ref.)
	Second line	1 (Omitted)	1 (Omitted)	1 (Omitted)	1 (Omitted)
Viral load suppression	No	1 (Ref.)	1 (Ref.)	1 (Ref.)	1 (Ref.)
	Yes	1.30 (1.27, 1.33) ***	1.41 (1.28, 1.54)***	1.43 (1.22, 1.67)***	1.27 (1.10, 1.47)**
AIC		125.34	126.27	122.65	118.35

* = P-value <0.05

** = P-value <0.01

*** = P-value <0.001

AIRR = Adjusted Incidence Rate Ratio

## Discussion

Antiretroviral treatment began in 2003 and free ART was launched in Ethiopia in 2005. An estimated 738,976 Ethiopians are currently living with HIV and all of them require antiretroviral treatment (ART). However, only 426,000 are currently taking ART [21]. Monitoring individuals receiving ART is important to ensure successful treatment, identify adherence problems and determine whether and which ART regimens should be switched in case of treatment failure [21]. This retrospective cross-sectional study was conducted to estimate the proportion of patients with immunological recovery, failure and to identify factors associated with recent CD-4 count progression over time after ART commencement.

The median baseline CD4+ count of this study was 201cells/μL. This finding was similar to a study conducted in Jimma, Ethiopia, which was 191 cells/μL [22]. However, the current finding was higher compared with studies conducted in the capital city of Addis Ababa, which were 115 cells/μL [23] and 177 cells/μL [24]. Similarly, lower median CD4+ counts were reported from the Tigray region (162 cells/μL) [17], Felege-hiwot referral hospital, Bahir Dar, Ethiopia (147 cells/μL) [25], southern Ethiopia (156 cells/μL) [26] and Kenya (152 cells/μL) [27]. Nonetheless, a study conducted in Liberia revealed a higher median CD4+ count (238 cells/μL) [28]. This variation may be explained due to differences in the minimum standards of CD-4 count to initiate ART treatments and delayed initiation of ART. This could also be attributed due to the poor awareness of the public.

The proportion of patients with a baseline CD-4 count of <200 cells/ mm^3^, 200–499 cells/ mm^3^ and ≥ 500 cells/ mm^3^ was 49.61%, 38.95% and 11.44% respectively in this study. A study conducted in Somali region, Ethiopia showed that 57.6% of the patients had a baseline CD4+ count ≤ 200 cells/ mm^3^ and 42.4% had >200 cells/ mm^3^ [29]. Similarly, about 15.59%, 45.63% and 38.79% of the participants in this study had a recent CD-4 count of < 200 cells/ mm^3^, 200–499 cells/ mm^3^ and ≥500 cells/ mm^3^ respectively. A study conducted at the Felege Hiwot Referral Hospital reported that 30.8% of the patients had recent CD-4 count of ≥ 500 cells/ mm^3^ [30]. With regard to achieving a normal CD-4 Count (≥ 500 cells/ mm^3^), the current study was similar to the study conducted in the Felege Hiwot referral Hospital where 37.6% of them reached a CD4 count of 500 or more cells/mm^3^ [31], while other studies reported a proportion of 45.2% [32] and 59% [33] of the patients achieved normal range of CD-4 count. Potential explanations for the differences could be that Ethiopians might have a relatively lower normal CD4 count compared to other study populations, though it requires verification, differences in the duration of ART, epidemiology of opportunistic infections, age of study participants, and methods used to determine the CD4 count (5, 10, 12, 34). The median (±IQR) recent CD-4 count was 423 (±347) cells/ mm^3^. This was lower compared to the mean CD4 count of healthy HIV negative Ethiopians which was 820 (±270) [34]. This indicates that the difference in the duration of ART might not be enough to achieve CD-4 count plateau.

Immunologic recovery was attained by only 28.35% of the patients, which was lower when compared with another study 45.2% [32]. This study has revealed immunological failure in 2.14% and 19.42% of the patients based on WHO 2016 and 2013 definitions respectively [11, 12]. Considering the recent WHO definition, this finding was lower compared with the study conducted in Felege-hiwot hospital Bahir Dar, Ethiopia (15.9%) [25], 6.5% in Tigray region [17], 15.7% in Federal Police Referral Hospital (FPRH) Ethiopia [23], 13.2% in Gondar referral Hospital [16], 6.1% in the Somali region of Ethiopia [29]. However, based on the findings of the WHO definition 2013 immunological failure were higher compared to different studies [16, 17, 23, 25, 29]. These variations might be due to the application of different WHO definitions, which was changed over time or it could be a real difference.

Good adherence compared to poor adherence was positively associated with CD-4 count in this study. Similar studies from Ethiopia, Kenya, Colombia and France have reported that patients with non-adherence to treatment were associated with immunological failures when compared with good adherence [17, 27, 29, 35, 36]. This can be justified, in the case of non-adherence, the level of antiretroviral drug concentration in the blood might not suffice to suppress the viral RNA replication and this in turn deplete CD-4 T-cells in the blood.

Higher baseline CD4 count was positively associated with the recent CD-4 count during treatment. Different studies have also supported this finding that higher baseline CD4 count was positively associated with the time to immunologic recovery [30, 31]. Another study has also reported a baseline CD4 count was positively associated with the recent CD-4 count [37]. However, there were contradicting studies which reported a negative association between baseline CD4 count and CD4 count during the treatment period [38, 39]. In fact, the nature of the parameters estimated in these contradicting studies is different. The current study measured the gain in CD4 count from each baseline CD4 count without considering the time variation from initiating the ART; but these studies which reported negative associations estimated the average rate of increment or slope per unit time (considering time variable) which could diminish over time until it reaches a plateau.

Age 15–19 years was positively associated with an increase of the recent CD4 count. There was some evidence which showed that commencing HAART at a younger age may be associated with an improved immunologic response [40, 41]. On the contrary, other studies reported a null association between age and CD4 count increment [32, 38]. These studies have a small sample size [32, 38] which may not ensure homogeneity of different age groups.

Females compared to males have positive associations with the recent CD-4 cell count. This finding did not agree with the finding [31], however, this finding agrees with different studies [38, 42, 43]. A similar study has also shown that females had better response to ART as compared to males [43]. This can be justified as females could attend voluntary counseling as part of their routine health care services during pregnancy, while male patients are poor in their health seeking behavior as they experience lower rates of HIV testing, and acceptance of linkage to HIV-care after a positive result [44]. As a result, females are more likely to be diagnosed in HIV infection earlier than males [45,46], and this could make the response to ART treatment poor for lately diagnosed males as the immune system could be damaged irreversibly in advanced stages of the disease [47]. Therefore, health seeking behavior and early presentation might have been different in males and females.

This study showed that patients in WHO stage II and III were negatively associated with increment in the recent CD-4 count compared with the WHO stage I. Different other studies have also shown that WHO clinical stage and classification of HIV/AIDS correlates well with CD4+ T-lymphocyte counts [48, 49]. Although WHO stage IV is the reason for poor immunological recovery from the science of knowledge, however, WHO stage IV was not statistically associated for recent CD-4 count in this study. The absence of statistical association might partly be attributed due to low sensitivity of WHO clinical stage in predicting CD-4 count [50] and/or it could be a real scenario. The other reason might be, about 44.81% and 1.91% of the patients with less than 200 CD-4 T-cells/ mm^3^ in this study were classified in WHO stage I and II respectively. However, patients with <200 CD-4 T-cells count are more likely to have advance HIV infection that might be classified into WHO staging IV or III. This shows that there was wrong way of classifying patients into WHO stages at baseline and hence affects the association.

Patients with viral suppression were positively associated with the recent CD-4 T-cells count. A similar other studies from Ethiopia, Nepal, Thailand and India reported that patients with HIV RNA level ≥1000 copies/ml were more likely to experience immunological failure in ART follow up as compared with those who had an HIV RNA level less than 1000 copies/ml [17,51,52,53]. Another study has also indicated that maintaining virological suppression results in greater increases in CD4 cell counts in the long term [54]. This is because as the load of viremia decreases, the depletion of CD-4 cells might be decreased.

Patients from military health care facilities had a higher CD-4 cells count as compared to patients from non-military health care facilities. Even though there were no similar studies which support or contradict this finding, patients from military health care facilities were mostly military staff that might have relatively higher adherence to treatment and nutritional status as compared to civil patients from non-military facilities which then results in a boost of CD-4 cells count after Antiretroviral treatment.

Patients with a first line regimen 1e (TDF-3TC-EFV), and second treatment line regimens 2f (AZT-3TC-ATV/r) and 2h (TDF-3TC-ATV/r) were negatively associated with recent CD-4 count as compared to 1c (AZT-3TC-NVP). Though there were no similar studies in relation to recent CD-4 count and antiretroviral regimens, there was reported finding that initiating ART using any one of the following ART regimens: 1c (AZT-3TC-NVP), 1d (AZT + 3TC + EFV) and 1e (TDF + 3TC + EFV) prevented treatment failure [51]. Even though there is concordance with 1c, the variation with other regimes might be due to the small size of sample size in the later study, which might not insure the accuracy by avoiding sampling errors such as large variability, bias or under coverage.

Compared to routine first Viral Load (6 months or more on ART); suspected ART failure initial Viral Load, suspected ART failure immunological and suspected ART failure clinical were negatively associated with the recent CD-4 count. There was a similar finding from Uganda, where being a suspected treatment failure patient and repeat test after suspected failure were positively associated with viral non suppression [55]. This can be justified, as HIV viremia increases the probability of CD-4 T-cells depletion might increase.

### Strength and limitations of the study

This study was not without limitation. Due to the nature of secondary data, there were no data when the baseline and recent CD-4 T-cells count was conducted and hence the time when the immunological failure/ recovery had occurred is not known. In addition to the time of ART exposure the analysis misses some important variables such as the existence of co-infection and grade of ART experience in the HIV infected patients. Despite these limitations, this study was done on a large sample size with appropriate statistical analysis techniques that provides important information regarding viability of ART treatment program in Tigray, Northern Ethiopia.

## Conclusions

Immunological recovery was lower, only one third of the patients achieved immunological recovery despite being on highly active ART (HAART). Sex, CD4 baseline, age, ART clients served in defense facility, WHO Stages II and III, adherence, virological test reasons of suspected ART failure clinical, suspected ART failure immunological and suspected ART failure initial Viral Load, regimen type of 1e, 2f and 2h and viral load suppression were significantly associated with CD-4 T-cells progression over time, among HIV patients on ART in Tigray region. Therefore, intensive adherence counseling, follow-up and support should be provided focusing on patients with virally non suppressed individuals so as to enhance immunological recovery. In addition, determinants of immunological recovery need to be investigated in detail to design an appropriate intervention.
